# HIV-1 vaccine immunogen design strategies

**DOI:** 10.1186/s12985-014-0221-0

**Published:** 2015-01-24

**Authors:** Jaclyn K Mann, Thumbi Ndung’u

**Affiliations:** HIV Pathogenesis Programme, University of KwaZulu-Natal, 719 Umbilo Road, Durban, 4001 South Africa; KwaZulu-Natal Research Institute for Tuberculosis and HIV, University of KwaZulu-Natal, Durban, 4001 South Africa; Ragon Institute of MGH, MIT and Harvard University, Cambridge, MA 02139 USA; Max Planck Institute for Infection Biology, Chariteplatz, D-10117 Berlin, Germany

**Keywords:** HIV-1 vaccine, Immunogen design, Broadly neutralising antibodies, T cell immunogens

## Abstract

An effective human immunodeficiency virus type 1 (HIV-1) vaccine is expected to have the greatest impact on HIV-1 spread and remains a global scientific priority. Only one candidate vaccine has significantly reduced HIV-1 acquisition, yet at a limited efficacy of 31%, and none have delayed disease progression in vaccinated individuals. Thus, the challenge remains to develop HIV-1 immunogens that will elicit protective immunity. A combination of two independent approaches - namely the elicitation of broadly neutralising antibodies (bNAb) to prevent or reduce acquisition of infection and stimulation of effective cytotoxic T lymphocyte (CTL) responses to slow disease progression in breakthrough infections (recent evidence suggests that CTLs could also block HIV-1 from establishing persistent infection) – is the current ideal. The purpose of this review is to summarise strategies and progress in the design and testing of HIV-1 immunogens to elicit bNAb and protective CTL immune responses. Recent advances in mimicking the functional native envelope trimer structure and in designing structurally-stabilised bNAb epitope forms to drive development of germline precursors to mature bNAb are highlighted. Systematic or computational approaches to T cell immunogen design aimed at covering viral diversity, increasing the breadth of immune responses and/or reducing viable viral escape are discussed. We also discuss a recent novel vaccine vector approach shown to induce extremely broad and persistent T cell responses that could clear highly pathogenic simian immunodeficiency virus (SIV) early after infection in the monkey model. While *in vitro* and animal model data are promising, Phase II and III human clinical trials are ultimately needed to determine the efficacy of immunogen design approaches.

## Background

**Figure 1:**
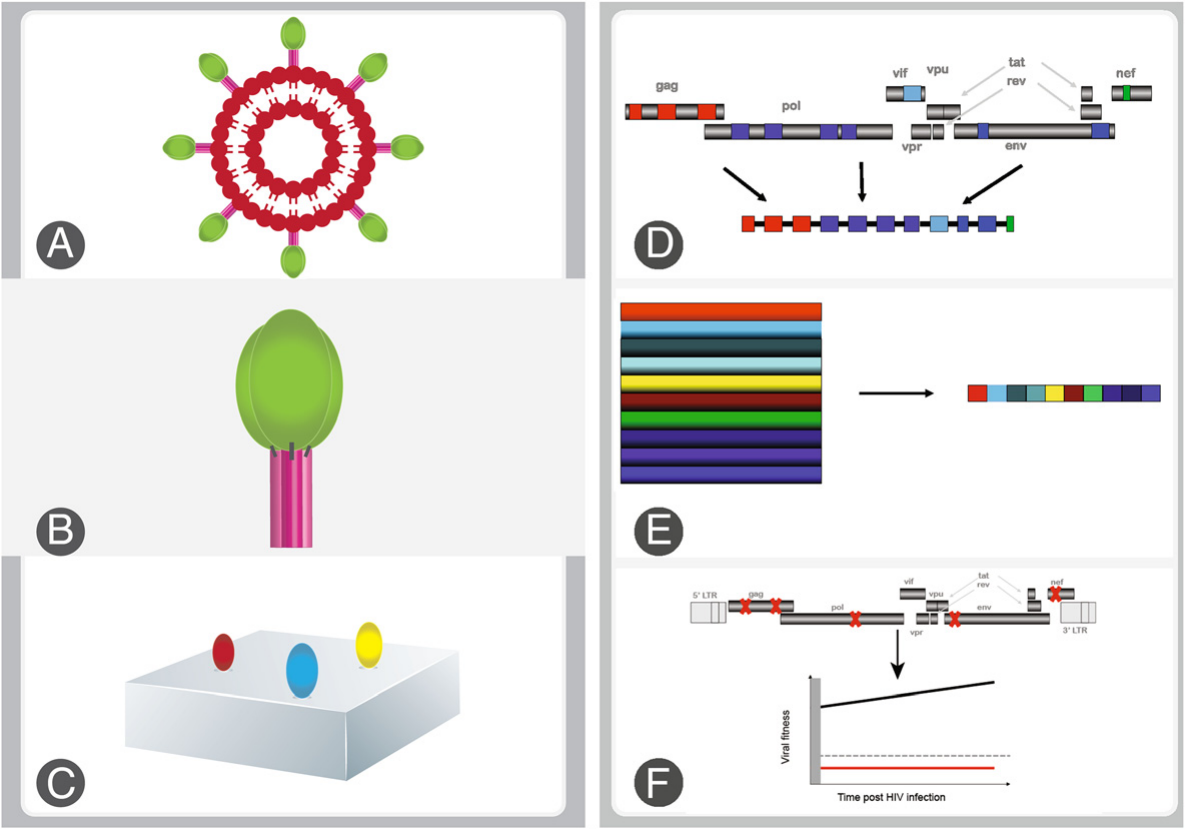
**Neutralizing antibody and T cell based immunogen design strategies.** In this schematic, some immunogen design strategies discussed in this review are highlighted. **A)** A virus-like particles (VLP) from which non-functional Env have been removed from the surface by enzymatic digestion is shown. Gp120 and gp41 native trimers are represented in green and pink respectively. **B)** A native stabilised soluble envelope trimer is shown. Current strategies for stabilisation include mutation of the gp120-gp41 cleavage site, introduction of trimerisation domains, introduction of disulphide bonds between gp120 and gp41, and gp41 trimer stabilising mutations. The modifications (represented by black solid lines) may enhance solubility, reduce aggregation or expose neutralizing antibody epitopes. **C)** Stabilization of neutralizing antibody epitopes- represented by the red, blue and yellow ovals- on a molecular scaffold (grey) after they have been identified by mutagenesis and computational approaches. **D)** Mosaic immunogens comprise a small number of protein sequences from various HIV proteins and are created using computational approaches from recombination of naturally-occurring protein sequences in a given viral population. They are designed to achieve maximal coverage of natural variation of all potential T cell epitope sequences in a particular viral population. **E)** T cell epitopes and conserved elements are identified and used to construct an immunogen which may also require optimisation for expression, processing and antigen presentation. **F)** Immunogens designed to impact on viral fitness and prevent viral immune escape. In this approach, immune responses are targeted at epitopes in which escape results in significant fitness cost on the virus, or target compensatory mutations (both depicted by a red crosses). The virus is thus suppressed by effective T cell immune responses or becomes attenuated following escape. The attenuated virus has very low in vivo fitness (shown by the red line) and cannot be transmitted or cause disease following acute HIV infection (grew window). The grey broken line represents a hypothetical fitness threshold that would need to be achieved for this strategy to work.

It is estimated that 35.3 million people were living with human immunodeficiency virus type 1 (HIV-1) at the end of 2012, and the epidemic continues to spread, with approximately 2.3 million new infections diagnosed in 2012 [1]. Although much progress has been made in the development of other biomedical prevention modalities, an effective prophylactic HIV-1 vaccine is expected to have the greatest impact on HIV-1 spread [2]. The challenges to developing an HIV-1 vaccine are numerous. A completely protective HIV-1 vaccine would have to induce effective immune responses to clear the infection within the narrow window of a few days before a latent reservoir is established [3,4]. Further, these immune responses would have to be effective against the enormous diversity of HIV-1 strains, which far exceeds the diversity of influenza for which a new vaccine is needed every year [5], and the high mutability of the virus results in ease of escape from immune responses, generally with immune responses lagging behind virus evolution [3,4]. Broadly neutralising antibodies (bNAb) have the potential to provide complete protection from infection, however there are considerable challenges in inducing these antibodies by vaccination. The conserved epitopes to which they are directed are masked and poorly immunogenic, and these antibodies are generally characterised by high levels of somatic hypermutation and a long heavy chain complementarity-determining region 3 [HCDR3] (although, the recent observation that long HCDR3 are a normal part of the human naïve B cell repertoire and understanding of the immunologic basis of long HCDR3, indicates that this may not be an insurmountable hurdle [6]), suggesting that complex maturation pathways are required [2,6-8]. Furthermore there is a lack of knowledge of the correlates of protection and available animal models are not necessarily reliable in predicting outcomes in humans [9]. Figure 1 is a summary of approaches currently being pursued in HIV vaccine design, based on immunogens that may elicit broadly neutralising antibodies and effective antiviral T cells.

Thus far, there have been more than 218 HIV vaccine trials, but only 5 vaccines have advanced to Phase IIb and III clinical trials, including the VAX003, VAX004, Step/Phambili, RV144 and HVTN505 trials [2,10,11]. The VAX003 and VAX004 Phase III trials tested bivalent recombinant HIV-1 envelope (Env) gp120 B/E and B/B vaccines, respectively, however they induced non-neutralising antibodies and showed no significant reduction in HIV-1 acquisition in the vaccinated populations [12,13]. The Step (conducted in America and Australia) and Phambili (conducted in South Africa) Phase IIb trials evaluated the efficacy of a vaccine comprising adenovirus serotype 5 vector expressing the HIV-1 *group specific antigen (gag)*, *polymerase (pol)*, and *negative regulation factor (nef)* genes, but these trials failed to show a protective effect, which may be related to non-optimal specificity and breadth of CD8+ T cell responses elicited [14]. The most recent vaccine regimen to undergo Phase IIb testing (HVTN505 trial) consisted of a deoxyribonucleic acid (DNA) vector encoding HIV-1 clade B Gag, Pol, and Nef proteins and multi-clade Env proteins followed by a recombinant adenovirus type 5 boost [15]. This vaccine failed to have any significant effect on HIV-1 acquisition or on viral load in infected vaccinees. Thus far, only one candidate vaccine - a recombinant HIV-1 (*env-gag-protease*)-canarypox vector prime and a recombinant gp120 plus alum boost tested in the RV144 Phase III trial - has impacted significantly on HIV-1 acquisition, and this was at a limited efficacy of 31% [16]. Subsequent analyses have identified Env V1-V2 immunoglobulin (Ig) G antibodies as a correlate of reduced risk of HIV acquisition and IgA Env antibodies as a correlate of risk in this trial, and there is evidence that the antibody-dependent cell-mediated cytotoxicity (ADCC) activity of antibodies may have contributed to the protective effect [17,18].

Following these trials, the current thinking is that an approach using two independent, non-interfering and complementary vaccines – one designed to initiate bNAb (and/or ADCC activity) with the aim of blocking/reducing acquisition of infection, and one designed to elicit effective cytotoxic T lymphocyte (CTL) immune responses to control breakthrough infections – may be preferable [4,19]. Furthermore, since empirical approaches to developing an HIV vaccine have had little success thus far, efforts are focussed on rational approaches to vaccine design, although some caution that these approaches should be pursued in conjunction with clinical trials as observations in humans are often unexpected and are valuable in informing vaccine development [11]. This review will focus on current systematic structural or computationally-driven approaches to the design of B cell and T cell vaccine immunogens (summarised in Table 1). Although vector choices and adjuvants (both of which modulate innate immunity that in turn shapes adaptive immunity [20]) as well as delivery systems critically influence immunogenicity and efficacy of immunogens [9,21], and constitute a separate part of vaccine design, these topics will not be discussed in this review.

**Table 1:** **Summary of immunogen design strategies and progress in evaluation**

**Design strategy**	**Expected outcome**	***In vitro*** **evaluation**	**Animal trials**	**Human trials**	**Key ref.**
1. Mimicking native trimer: remove non-functional Env from VLP	bNAb	Recognised by NAb but not non-NAb	-	-	[29,31]
2. Mimicking native trimer: soluble SOSIP-modified Env trimer	bNAb	Recognised by bNAb but not non-NAb Resembles Env trimer by electron microscopy	-	-	[39]
3. Stabilised bNAb epitope: epitope-scaffolds	bNAb	Bound to bNAb	Ones tested did not elicit NAb	-	[33,46,47]
4. Stabilised bNAb epitope: targeting germline and driving maturation	bNAb	Potently activated germline and mature VRC01 B cells	-	-	[49]
5. Stabilised bNAb epitope: fragment immunogen	bNAb	Ab induced in rabbits neutralised tier I, II and III viruses	Induced b12 bNAb in rabbits	-	[51]
6. Mosaic immunogens	T cell responses to diverse strains, reduce escape	Processed and expressed by human T cells	Increased breadth and depth of T cell responses Reduced per exposure probability of infection by ≈ 90%	-	[67,70-72]
7. Conserved element immunogens	T cell responses to diverse strains, reduce escape/attenuate virus	T cell responses elicited in humans inhibited viruses	Highly immunogenic	High magnitude and breadth of T cell responses in 100% vaccinees	[21,81]
8. Escape-cornering immunogens (computational model)	Reduce escape/attenuate virus	Fitness testing of mutants supported model predictions	-	-	[84,86]
9. Immunogens using CMV vectors	Persistent T cell responses to act early	-	50% monkeys clear SIV infection early Persistent, unusually broad T cell responses	-	[55,91,92]

ref – references; VLP – virus-like particles; bNAb – broadly neutralising antibodies; NAb – neutralising antibodies; SOSIP - disulphide bond between gp120 and gp41 and gp41 trimer stabilising mutation I559P; Env – envelope; CMV – cytomegalovirus; SIV – simian immunodeficiency virus.

### B cell immunogens

There is growing evidence that non-neutralising antibody functions contribute to viral control and protection from infection. Transactivator of transcription factor (Tat)-binding antibodies have been linked to viral control in HIV-1 infection [22] and ADCC activity, which is largely mediated by Env-binding antibodies [23], has been correlated with reduced viremia following simian immunodeficiency virus (SIV) challenge in macaques [24,25]. Furthermore, through passive immunisation of macaques with the b12 bNAb that was mutated to knockout Fc-mediated effector functions, it was demonstrated that non-neutralising activities of this antibody contributed to protection against simian/human immunodeficiency virus (SHIV) challenge [26]. There is also evidence that ADCC activity reduced risk of HIV-1 acquisition in the RV144 trial [17,18]. Although induction of effective non-neutralising antibody functions (and non-Env antibodies) may be a relevant component of a protective HIV-1 vaccine and requires further research, only neutralising antibodies have been directly and clearly shown to block virus transmission - as evidenced by passive transfer of bNAb to macaques followed by protection from infection with a SHIV challenge [27], and protection from HIV-1 infection in the humanised mouse model by use of vectors expressing bNAb [28]. Thus, current efforts, and the discussion in this review, are focussed on designing B cell immunogens that can successfully elicit bNAb. There are two major strategies being pursued to achieve this goal: creating immunogens that mimic (i) the native Env trimer and (ii) epitopes of bNAb stabilised on a structure.

### The native Env trimer

The presence of non-functional Env in natural infection is a decoy that results in immune-dominant non-neutralising antibody responses [29,30]. However, the natively folded functional Env trimer exposes neutralisation-sensitive surfaces while concealing surfaces targeted by non-neutralising antibodies [29] and is thus a promising vaccine immunogen for vaccines intended to elicit neutralising antibodies. Up until very recently it has been challenging to achieve the stable correctly-folded Env trimer form.

One recent approach has been to produce virus-like particles (VLP) and remove the non-functional Env from the surface by enzyme digestion [31] Figure 1A. This method effectively results in VLP bearing pure native trimers only, as evidenced by neutralising antibody (Nab) recognition but not non-NAb recognition following digests [29]. These digested VLP have yet to be fully evaluated for their potential to elicit neutralising antibodies.

There has also been an advance in the development of soluble Env trimers in the native form. Soluble Env trimers are not stable due to metastable gp41 and non-covalent interactions between gp120 and gp41 [32,33]. Several strategies have previously been employed in an attempt to stabilise soluble envelope trimers, including mutation of the gp120-gp41 cleavage site [34], introduction of trimerisation domains [34], introduction of disulphide bonds between gp120 and gp41 [35], and gp41 trimer stabilising mutations [36] (Figure 1B). However, immunization with cleaved soluble forms of Env trimers containing these modifications has yielded antibodies that are only slightly superior in terms of neutralisation potency and breadth when compared with monomeric gp120, and which generally only neutralised neutralisation-sensitive virus or autologous virus from the immunisation protocol [37,38]. A recent advance by Sanders *et al.* (2013) [32] is the engineering of a cleaved soluble stable Env trimer form with SOSIP modifications (disulphide bond between gp120 and gp41 and gp41 trimer stabilising mutation I559P) as before [36] but based on the clade A BG505 stain modified to introduce bNAb epitopes and truncated at residue 664 to enhance solubility and reduce aggregation [39]. This trimer form very closely resembles the native functional Env trimer by electron microscopy and mimics the antigenicity correctly (*i.e.* it reacts strongly with bNAb but not with non-neutralising antibodies) – thus it appears to be a very close mimic of the native Env trimer [39]. This BG505 SOSIP.664 Env trimer structure has recently been described at 4.7-5.8 angstrom resolution by x-ray crystallography [32] and cryo-electron microscopy [40]. This can provide detailed information about the presentation of bNAb epitopes in the native trimer context (previously information of the structure of these epitopes was derived from Env fragments, not in the full and proper antigenic context) which should be valuable to immunogen design based on these epitopes alone, as discussed in section 1.2 [32,40]. The BG505 SOSIP trimer might prove successful as a vaccine immunogen but this now has to be evaluated.

The whole Env trimer approach is however limited in the ability to direct responses to specific bNAb epitopes (these also tend to be immunorecessive), but the trimer approach could potentially be used as a boost in conjunction with constructs mimicking specific bNAb epitopes only [33,41]. It is also not known whether correct mimicking of the native Env trimer is sufficient to elicit bNAb, particularly since the development of bNAb requires that the Env first bind to the germline precursor of the bNAb followed by affinity maturation leading to the mature bNAb and evolved Env sequences generally do not bind to these bNAb germline precursors [42]. Thus the generation of bNAb may require immunisation first with specific Env epitope sequences that recognise the germline precursor, and then sequential immunisation with sequences that direct the affinity maturation process [43]. Next we discuss design of immunogens mimicking specific bNAb epitopes and strategies to achieve binding to germline precursors followed by affinity maturation to drive bNAb development.

### Stabilised bNAb epitopes

The first step in the design of bNAb epitope mimetics is the identification of bNAb epitopes. These may be identified using crystal structures of Env in complex with bNAb [32,44]. Recently, a computational method to predict the epitopes of bNAb - including non-linear epitopes - from viral sequences and cross-clade neutralisation activity data (these experiments are usually performed when bNAb are isolated) in the absence of structural information, has been developed [45]. This method accurately predicted the known key residues of several bNAb epitopes, as well as key epitope residues for two recently isolated bNAb which were subsequently validated experimentally [45]. Thus, this approach has the potential to speed up identification of bNAb epitopes.

Following identification, the goal is to create a mimic of the bNAb epitopes (Figure 1C). This is particularly challenging since bNAb epitopes are often non-continuous – they constitute regions brought together in three dimensional structure. However, recently, computational-guided methods have been used to design scaffolds onto which epitopes are grafted such that epitope presentation closely mimics the natural presentation in terms of structure [46,47]. Epitope-scaffolds have yet to elicit broadly neutralising antibodies, however improvements are in progress and neutralisation data has not yet been reported for some of the designs [33,47]. It is likely that several different scaffolds presenting the same epitope may have to be used in an immunisation strategy in order to avoid dominant immune responses to the scaffold itself rather than the epitope [33].

Generally, circulating Env forms do not bind to the germline precursors of bNAb [42,48], and thus efforts are being made to modify bNAb epitopes such that they can bind to germline receptors [48,49]. However, this approach has challenges because suitable germline precursors for a particular bNAb will not be present in all individuals and more than one bNAb is likely to be required for protection. A potential solution may be to attempt to induce several bNAb lineages with a vaccine [50]. However, there is particular interest in designing immunogens to bind to germline precursors of the VRC01 bNAb since these precursors are expected to be present in most individuals (VH1-2 genes, from which these antibodies are derived, are present in approximately 2% of the human antibody repertoire) [49]. Recently, selective disruption of glycosylation sites has been demonstrated to release the block in Env binding to germline precursors of VRC01 bNAb [48]. In another important study, optimisation of a gp120 outer domain construct (encompassing the VRC01 epitope) such that it bound germline precursors with moderate affinity and mature VRC01 with high affinity (creating an affinity gradient for somatic mutation towards maturation) was achieved through a combination of computational methods to identify mutations expected to improve antibody binding followed by *in vitro* screening of antibody binding activity [49]. The optimised construct was then multimerised on virus-like nanoparticles and in this form was able to potently activate B cells expressing germline or mature VRC01 [49]. This promising vaccine candidate, perhaps in combination with different immunogens designed to drive/complete the affinity maturation process [49], will yet be evaluated for the potential to elicit VRC01 bNAb. Important insights were also recently made through studying both viral Env evolution and the evolution of the CH103 bNAb from the time of infection through to the development of mature CH103 bNAb in one individual [43]. Such co-evolution studies could greatly inform the process of driving a particular bNAb lineage from the germline to maturity: one could first immunise with the transmitted Env epitope that bound to the germline precursor and then later immunise with a swarm of the subsequent variants that drove development of the mature antibody, or this could also be done in a sequential manner [43]. However, this approach has not yet been implemented and given the complexity of Env and bNAb evolution within an individual, it may prove challenging in practice.

The first induction of b12 bNAb in rabbits immunised with a fragment immunogen representing 70% of the b12 epitope in which b12 residues were connected by 4 linkers, was recently achieved [51]. The antibodies induced neutralised several tier I, II and III viruses (tier II and III viruses are more difficult to neutralise than neutralisation-sensitive tier I viruses) in a panel of 21 viruses [51]. This promising immunogen, which is currently undergoing further development and testing, importantly demonstrates that bNAb can be elicited in small animal models [51]. However, this is anticipated to be a problem for some bNAb as the correct germline precursors do not appear to be present in these animal models and thus immunogens designed to elicit those antibodies may require testing in humanised mice or humans [49].

It should be mentioned that recently a correlation between the frequency of CD4+ T follicular helper cells and the development of bNAb has been observed [52], and it is likely that stimulation of these cells (which provide specialised help to B cells) will be required for optimal generation of bNAb.

### T cell immunogens

bNAb are the ideal for providing sterilising immunity, but T cells play an important role in controlling viremia [53,54] and vaccines eliciting effective T cell responses have the potential to control viremia to low levels long-term [19], or even possibly eradicate infection if early, strong responses are induced [55]. Enormous viral diversity and the ease of viral escape from immune responses are the major challenges to T cell immunogen design. Also, it has been shown that breadth of T cell responses to Gag correlates with control [56,57], and the ability to make multiple T cell responses to conserved epitopes is beneficial [58]. However, T cell immunogen designs have so far been unsuccessful in conferring protection or in control of HIV-1 infection. For example, the HIVA immunogen based on HIV-1 subtype A consensus sequence was poorly immunogenic, perhaps related to the vector delivery system [59]. Thus existing approaches to immunogen design include mosaics proteins to cover diversity and induce broad responses (yet including variable protein regions) and immunogens based only on conserved/vulnerable regions, which should also match diverse strains as well as increase difficulty for viral escape. There are also antigen processing modification approaches that may be able to focus T cell responses to conserved regions and increase strength of immune responses [60]. Generally, these proposed immunogens are presented within the context of replicating or non-replicating viral vectors. Although vectors are outside the scope of this review, a novel and exciting T cell vaccine approach which deserves mention is the use of a persistent replicating viral vector that induces extremely broad and persistent CTL responses to the retroviral insert [4], and this will also be briefly discussed.

### Mosaic immunogens

There are several approaches aimed at overcoming HIV-1 diversity which all focus on using the most representative viral sequence of a population as an immunogen [61]. A natural sequence that is closest to the consensus sequence of the population may be selected as an immunogen [62]. On the other hand, a central sequence may be constructed such as a consensus sequence (the most common amino acid at each codon in an alignment of the population sequences) [63,64], ancestral sequence (the most common recent ancestor of the population of sequences reconstructed from a phylogenetic tree) [63,65], or ancestral centre-of-tree sequence (the position on a phylogenetic tree that represents the least summed distance to all population sequences) [66]. These central sequences are genetically more similar to circulating strains than any natural sequence, however the central sequences are artificial and thus it is possible that they may not result in natural conformations (an advantage of the natural sequence approach), although central sequences tested have been shown to be expressed, functional and are immunogenic in animals [61,63-66]. Mosaic immunogens, however, provide the best coverage of a population of sequences, and only comprise natural sequence stretches, thus we will discuss this approach in more detail.

Mosaic immunogens are comprised of a small number of mosaic protein sequences (*e.g.* Gag) which are created, using computational approaches, from recombination of naturally-occurring protein sequences in a given viral population (*e.g.* M group HIV-1 Gag sequences) and selected on the basis of together achieving maximal coverage of natural variation of all potential T cell epitope sequences (all 9-mer sequences) in that viral population [67] (see Figure 1D). A single mismatch in a T cell epitope has a high chance of conferring escape from that particular T cell response [68], and therefore mosaics are aimed at high coverage of perfect matches of 9-mer sequences to reduce likelihood of mismatches between the vaccine and infecting virus and minimise vaccine-specific T cell responses. Mosaics provide better coverage of a population of sequences than any single natural sequence or central sequence - mosaics based on M group sequences provide good coverage of all M group subtypes (*e.g.* M group Gag mosaics achieve 74% coverage of perfect matches for global Gag sequences) [67]. Thus M group mosaics are good candidates for a global vaccine. Mosaic proteins are designed such that they only contain naturally-occurring sequence stretches, avoid artificial junctional epitopes and represent full-length intact proteins; thus it is thought that they will be processed as in natural infection [61]. This has not been the case for polyepitope vaccine candidates (single epitopes linked together, with or without optimisation for antigen processing), which have demonstrated good immunogenicity in animal models yet extremely poor immunogenicity in human trials [61,69]. A study investigating the immunogenicity of a mosaic Gag, Pol and Env vaccine in rhesus monkeys showed that the mosaic vaccine considerably increased the breadth and depth (responses to more than one variant of an epitope), without affecting magnitude, of immune responses elicited when compared with vaccines based on natural or consensus sequences [70]. This vaccine also significantly reduced the per exposure probability (by approximately 90 of monkeys becoming infected following a stringent challenge with a neutralisation-resistant virus [71]. While it has been demonstrated that mosaic antigens are processed and expressed by human T cells *in vitro* [72], immunogenicity trials in humans are necessary. Phase I clinical trials of mosaic vaccines are in the pipeline [73].

### Conserved elements immunogens

Another proposed T cell vaccine strategy which also tackles the issues of addressing global diversity and the ability of HIV-1 to escape immune responses, is to design immunogens restricted to only the most conserved regions of HIV-1 (Figure 1E). T cell escape mutations in conserved rather than variable regions tend to carry significant fitness costs [74]; therefore a vaccine targeting multiple conserved epitopes and excluding variable regions has the potential to limit immune escape or attenuate HIV in the event of immune evasion [75-77]. In natural HIV infection the immunodominant T cell responses, that largely absorb the T cell arm of the immune system and suppress the subdominant responses, are usually to variable epitopes that escape easily with little fitness cost, while responses to conserved regions are typically subdominant [75,78]. Including only conserved elements in the vaccine immunogen avoids diversion of responses away from conserved regions by decoy immune responses to variable regions [75], and therefore has an advantage over the full-length mosaic protein approach: While full-length Gag mosaic proteins elicited comparable breadth and increased magnitude of responses to conserved regions when compared with the same mosaics restricted to conserved portions only in rhesus macaques, the full-length proteins also elicited relatively stronger responses to some of the more variable regions of Gag [79]. Furthermore, another study in rhesus macaques showed increased breadth and magnitude of responses to conserved Gag p24 regions elicited by a conserved Gag p24 region construct as compared to the full-length Gag protein [80]. Therefore, full-length mosaics could result in the outcompeting of responses to conserved regions by responses to variable regions, which are more easily escaped and likely to incur escape if the vaccine does not silence the infection first [19]. However, mosaics of conserved element immunogens to further improve population coverage are a possibility to be considered [19].

The conserved element construct that has been most extensively tested thus far, is comprised of the 14 most conserved subprotein regions of HIV and is based on the consensus sequence alternating between clades A, B, C and D for the 14 segments [81]. High immunogenicity of the construct was shown in animal models prior to testing in humans [81,82]. In human Phase I trials, the vaccine induced unprecedented high levels of T cell responses, in 100% of recipients, to conserved regions (median peak magnitude of >5,000 spot-forming units (SFU)/million peripheral blood mononuclear cells (PBMCs) for two of the regimens tested and responses to 8 naturally subdominant conserved epitopes on average) [21] in contrast to the relatively lower breadth and magnitude of responses initiated by the STEP vaccine trial (77 of recipients had detectable T cell responses, which were in the range of 163–686 SFU/million PBMCs, and the majority of these recipients responded to 2–3 epitopes) [83]. Furthermore, effector T cells elicited by the conserved vaccine inhibited virus replication in autologous CD4+ cells for a panel of viruses *in vitro* [21]. Thus, despite being an artificial immunogen, rather than a full-length protein comprising only natural sequences, the conserved vaccine was processed adequately and induced T cells that recognised HIV-1 infected human T cells. The durability of these responses is currently unknown and this conserved immunogen has yet to be evaluated in Phase II trials.

### Escape-cornering immunogens

Related to the conserved vaccine strategy is the concept of an immunogen that blocks escape or corners the virus through exploiting antagonistic T cell escape pathways that may not occur simultaneously without substantial viral fitness costs [76,84,85] See Figure 1F). Recently, a computational model (applied to the HIV-1 Gag protein) was developed that predicts viral fitness based on sequence data alone, and can predict the fitness consequences of specific mutations alone or in combination and the fitness of any given viral strain based on its sequence [84,86]. *In vitro* experiments performed to test the fitness consequences of multiple mutations in HIV-1 Gag correlated strongly with model predictions, supporting this as a tool to design vaccine immunogens exploiting combinations of residues that are substantially fitness-reducing or non-viable when mutated simultaneously [84,86]. The immunogen design strategy proposed based on these fitness landscape models is to evaluate immunogens of all possible epitope combinations in a given protein and select the best one according to the following criteria: the fitness impact of simultaneously targeting epitopes within the immunogen, the fraction of the population that can target epitopes in the immunogen based on human leukocyte antigen (HLA)-I, and the number of epitopes in the immunogen [84]. Optimal Gag immunogens designed by these methods will yet be evaluated in animal models and may need to be extended to the entire HIV-1 proteome [84].

### Optimisation strategies for conserved region and escape-cornering immunogens

While immunogen strategies based on sequence conservation (section 2.2) or probability of observing a given sequence (section 2.3) may overall be reflective of viral fitness constraints, a few recent studies have indicated that there is not a transcendent link between sequence conservation and viral fitness [87,88]. Mutations at residues >98% conserved often result in substantial fitness costs but this is not always the case [87,88], and is less often true for highly conserved sites in Env than those in Gag p24 [88]. Therefore, ideally, immunogens designed based on frequency of mutations alone could be further evaluated in functional studies to fully describe fitness constraints and optimise design.

Another strategy proposed for optimising immunogens designed to elicit T cell immune responses to the conserved, vulnerable protein regions that are usually targeted subdominantly, is to selectively introduce sequence modifications that increase the yield of these particular epitopes following antigen processing [60]. It was recently observed that HIV-1 has adapted at the population level to common HLA-I variants by mutations that affect antigen processing and reduce the number of epitopes [60]. Since epitope abundance correlates positively with the magnitude and frequency of CTL responses elicited [60,89], artificial substitutions that increase the epitope abundance for epitopes restricted by common HLA-I variants in vulnerable regions could theoretically reverse the poor immunogenicity of these epitopes and refocus immune responses on these naturally subdominant regions [60]. A somewhat related optimisation strategy involves codon modifications (in the immunogen) to increase expression of the immunogen within a particular vector, thereby enhancing immunogenicity [90].

### Immunogens using cytomegalovirus (CMV) vectors

A persistently replicating rhesus CMV vector encoding SIV genes *gag*, *regulator of virion protein (rev)*, *tat*, *nef*, *env* and *pol* is the first vaccine that has been able to clear SIV infection in rhesus macaques by T cell responses [55,91]. The vaccine elicits persistent robust SIV-specific effector memory CTL responses (in the absence of neutralising antibodies), which are present to act immediately following infection, and results in 50% of vaccinated monkeys clearing SIV soon after infection, the protection being correlated with magnitude of peak SIV-specific CTL responses in the vaccine phase [55,91]. Another unusual feature, which could contribute to or even be responsible for the protection, is that SIV-specific T cell responses elicited are extremely broad (mean of 34 Gag epitopes covering 66% of the protein) and unconventional (they are largely HLA-II-restricted), which is an effect attributed to the modified rhesus CMV vector lacking 3 genes [92]. More recently, the HLA-I restricted T cell responses elicited by the vector were described to be largely of the HLA-E type, which is observed to be upregulated on SIV-infected cells – this unusual feature may therefore also partly explain the protective effect [93]. The next step is to generate human CMV/HIV immunogens designed to attenuate CMV and to address safety issues related to the use of a live CMV vector [4].

### Immunogen comparison and advancement through the vaccine pipeline

In addition to the scientific challenges in HIV immunogen design strategies, there is currently no consensus regarding practices and standards for comparative studies that may inform which designs should advance through the vaccine pipeline from preclinical research and development to efficacy trials. This is a major challenge for the field because specific standards for moving immunogens through the vaccine pipeline could save time and money. Additionally, the fact that i*n vitro* data on immunogen effectiveness may not always predict *in vivo* efficacy, limitations of current animal models used in HIV vaccine research, the lack of known correlates of protection and paucity of guidelines on “acceptable” immunogenicity levels for advancement of candidate HIV vaccines all mitigate against a universally acceptable standard for decision making about whether to advance products or not. Current practice for moving immunogens through preclinical development heavily relies on strength of scientific data suggestive of biological effectiveness plausibility but this can sometimes be subjective. In clinical studies, product safety in trial participants is a non-negotiable criterion for advancement, and this is usually accompanied by predetermined immunogenicity criteria. In both preclinical and clinical development of immunogens, the ease of product development or manufacture and the overall cost of moving from one stage of the pipeline to the next may also be important considerations.

## Conclusion

In recent years significant progress has been made in rationally designing immunogens to elicit bNAb and effective T cell responses. Some of these immunogens have shown promise either by measures of *in vitro* neutralisation activity, *in vitro* viral inhibition or protection from progressive infection in animal models. However, beneficial effects of vaccines observed in animal models have not always translated into efficacy in humans. *In vitro* neutralisation activity is correlated with protection in macaques from SHIV challenge [94] and *in vitro* viral inhibition by CTLs predicts the rate of CD4+ T cell decline and viral load set point in HIV-1 infection [95], indicating that these *in vitro* assays may be good measures of efficacious antibody and T cell responses, respectively. In conclusion, ultimately the success of a particular immunogen can only be determined from efficacy trials in humans.

## Abbreviations

ADCC: Antibody-dependent cell-mediated cytotoxicity
bNAb: Broadly neutralising antibodies
CMV: Cytomegalovirus
CTL: Cytotoxic T lymphocyte
DNA: Deoxyribonucleic acid
Env: Envelope
Gag: Group specific antigen
HCDR3: Heavy chain complementarity-determining region 3
HIV-1: Human immunodeficiency virus type 1
HLA: Human leukocyte antigen
Ig: Immunoglobulin
NAb: Neutralising antibodies
Nef: Negative regulation factor
PBMC: Peripheral blood mononuclear cells
Pol: Polymerase
Rev: Regulator of virion protein
SFU: Spot-forming units
SHIV: Simian/human immunodeficiency virus
SOSIP: Disulphide bond between gp120 and gp41 and gp41 trimer stabilising mutation I559P
Tat: Transactivator of transcription factor
VLP: Virus-like particles
